# Subcutaneous lateral brow lift (“Z-lift”)

**DOI:** 10.3205/iprs000075

**Published:** 2015-12-15

**Authors:** Klaus Ueberreiter, Ursula Tanzella, Yves Surlemont, Björn Dirk Krapohl

**Affiliations:** 1 Park-Klinik Birkenwerder, Germany; 2 Clinique Saint-Antoine, Rouen, France; 3 Department of Plastic and Hand Surgery, St. Marien-Krankenhaus Berlin, Germany; 4 Charité – Medical University of Berlin, Germany

**Keywords:** brow lift, aesthetic surgery, facial surgery

## Abstract

Surgical eyebrow lift has been described by using many different open and endoscopic methods. Difficult techniques and only short time benefits oft lead to patients’ complaints. We present a safe and simple temporal Z-incision technique for eyebrow lift in 37 patients. Besides simplicity and safety, our technique shows long lasting aesthetic results with hidden scars and a high rate of patient satisfaction.

## Zusammenfassung

Operative Augenbrauenhebung ist mit verschiedensten Methoden in offener und endoskopischer Technik beschrieben. Komplizierte Technik und Resultate, die oft nur von kurzer Dauer sind, führen zu Beschwerden der Patienten. Wir stellen eine sichere und einfache Technik mit temporaler Z-Inzision bei 37 Patienten vor. Neben Einfachheit und Sicherheit zeigt unsere Technik ein langzeitiges ästhetisches Ergebnis mit versteckten Narben und einer hohen Patientenzufriedenheit.

## Introduction

For many years, endoscopic forehead lift was the preferred tool and a plastic surgeon’s hand. But in many cases the position of the eyebrows especially in the medial part was relocated far too high. In a youthful appearance, usually the medial part of the brows is quite small as the lateral part is directed more upwards [1], [2], [3], [4], [5], [6].

Many patients who ask for an upper eyelid surgery show signs of lowered lateral brows. 

We developed a technique to lift those brows in a very easy and reliable way with excellent control of the appropriate vector with the necessity of basic instrument use only.

## Material and method

Since 2012 we have established the Z-lift routine procedure, meanwhile introduced in about one third of all our brow lifts. So far 37 patients have been evaluated. 

The procedures were usually combined with an upper eyelid blepharoplasty and also with facelift procedures, hence avoiding a cranial scar extension.

Only in 13 cases the Z-lift was performed as a stand-alone procedure. The effect of the lift was measured by photographic means six months after the operation. Patients were also asked about their satisfaction with the results.

### Planning of operation

**Figure 1:**
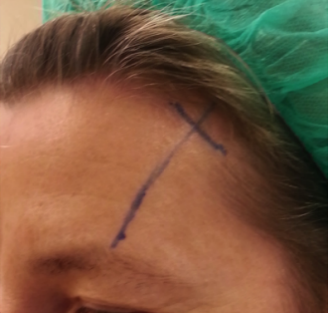
Marking of the vector for the eyebrow elevation

Before the intervention the vector of the planned brow lift is checked by pulling up the skin with one hand. The patient is invited to check the effect in the mirror. The resulting vector ranges between 30 and 70°. The vector line is drawn crossing the temporal hairline and a few centimetres behind this hairline, above the temporal muscle, a rectangular line is drawn to reside in a cross (Figure 1 (Fig. 1)).

**Figure 2:**
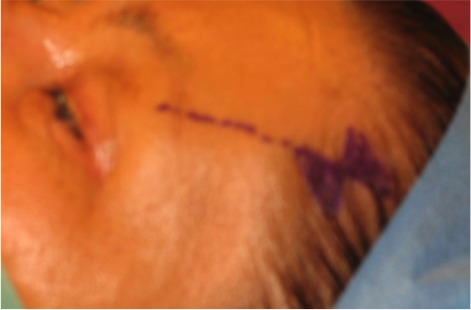
Marking of the hourglass shaped skin excision posterior to the hair line

The arms of this cross will be limited by markings to each one 1 cm length. In this way a shape like a small hourglass is created (Figure 2 (Fig. 2)). 

### Operation

The operation can be done under local anaesthesia, sedation, or general anaesthesia according to the patient’s and surgeon’s choice.

**Figure 3:**
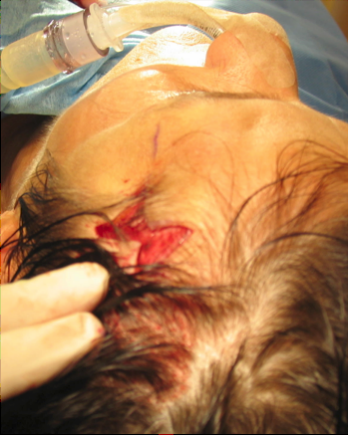
Temporal region after skin excision

**Figure 4:**
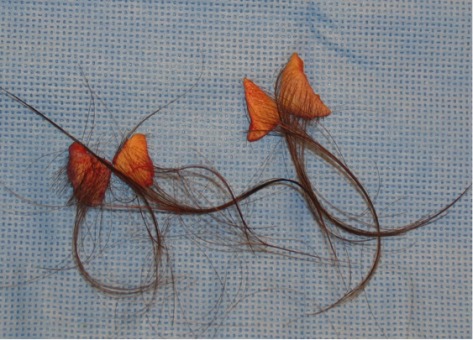
Resected skin, two triangular full thickness slides per side

**Figure 5:**
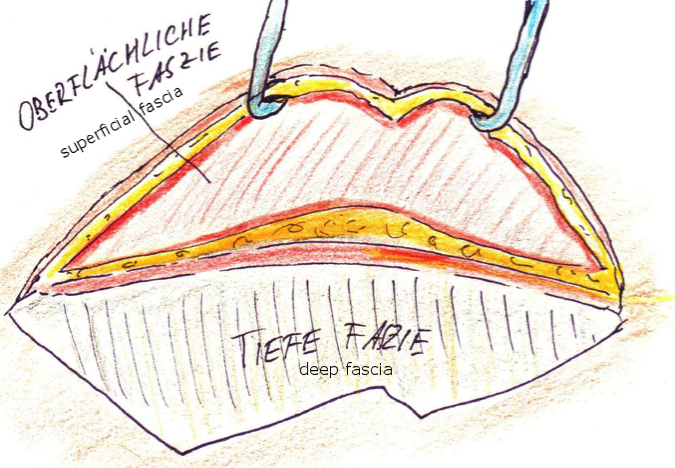
Temporo-frontal dissection, beginning strictly under the superficial temporalis fascia

The area around the hourglass drawing, along the vector, up to the medial part of the brows, going well to the lateral hairline and below the “crow’s feet” is infiltrated with local anaesthetic and epinephrine, usually about 30 to 40 ml per side. In the next step the complete excision of the upper and lower triangle of the hourglass is performed (Figure 3 (Fig. 3), Figure 4 (Fig. 4)), followed by incision along the line crossing the vector down to the space between the outer and inner sheet of the temporal muscle facia. The preparation follows this open space for roughly 1 cm in direction to the brows. Then a sharp hook is placed to elevate the skin with the adherent superficial fascia, and using a small blade, this fascia is cut in a semi-circular way to open up again the subcutaneous layer (Figure 5 (Fig. 5)). 

The preparation now follows exactly in the subcutaneous area until well below the brows and the lateral wrinkles.

The preparation should be carried out carefully to preserve the perforating vessels in the central part of the brows. It is finished, when an upward pull in the cross area easily lifts the whole undermined area. 

**Figure 6:**
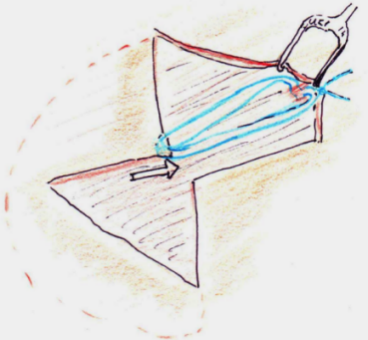
The actual brow elevation is achieved by a vicryl suture attaching the temporalis fascia flap cranially.

In the upper part now some centimetres in cranial direction on top of the lower temporal fascia are mobilized. The fascial patch adherent to the skin is now pulled up and attached with two 2-0 Vicryl sutures to the deep temporal fascia, cranially to the cross incision (Figure 6 (Fig. 6)). 

The brow is now lifted in a way that looks overdone, but within the next 3 to 4 weeks it will regain natural appearance. This over-correction is necessary to maintain a good permanent result. For skin closure we use staples. If still some bleeding occurs, a strip of latex or silicone can be inserted as a little drainage. Before applying the dressings, haematoma can be squeezed out and then immediately afterwards an elastic circular compression bandage is applied. This will be left in place for two days. The staples can be removed after 10 days. 

## Results

32 of our 37 patients rated the result as very satisfying or satisfying, three patients were disappointed with the amount of brow lift, and one was complaining about the small local baldness. Transient paralysis of the frontal branch of the facial nerve was observed in three patients. It resolved after 3 to 6 months and did not lead to complaints, because the brows are well attached in their new cranial position and the folds of the forehead had disappeared on the concerned side. The problem is always the over-reacting contralateral side which leads to an asymmetric appearance. This can be easily treated by injections of botulinum toxin.

Immediate post-operative haematoma occurred in five cases and could easily be resolved. With the learning curve of the surgeon this complication becomes very rare.

**Figure 7:**
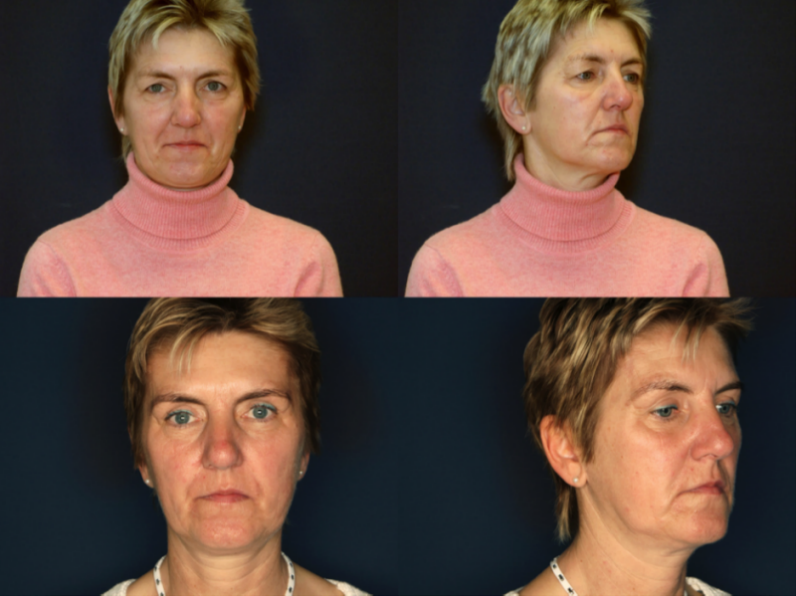
Patient one year after lateral Z-brow lift

**Figure 8:**
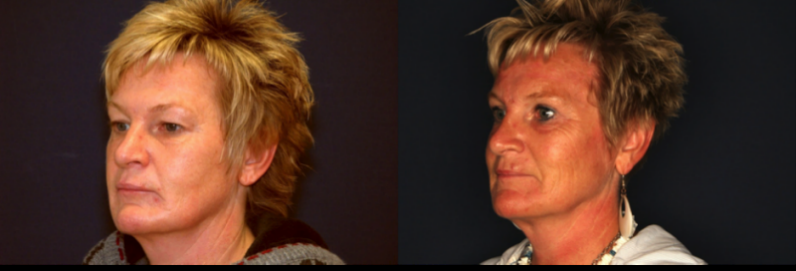
Patient 10 months after Z-brow lift

We present clinical examples of our results, see Figure 7 (Fig. 7) and Figure 8 (Fig. 8).

## Discussion

There are several common methods of eyebrow lift. The easiest approach is direct excision of skin above the brow line [7], [8], [9], [10], [11]. Another method is the approach via the blepharoplasty. The disadvantage hereby is the merely moderate short-time elevation of the brow.

**Figure 9:**
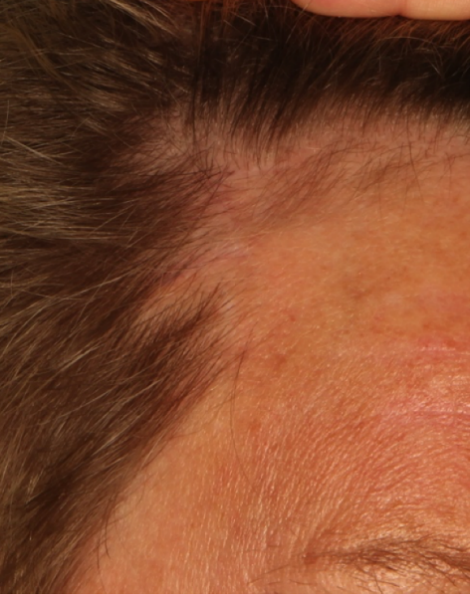
The scar is barely visible after 8 weeks and hidden behind the hair line.

The most common approach is the endoscopic lateral brow lift [12], [13], [14], [15], [16], [17], [18], [19], [20], [21], [22], [23], [24]. The disadvantage of this procedure is difficult stable anchoring and the complexity of the setup. Subcutaneous approaches have been described before [25], [26], [27], [28], [29]. We have developed a variation, which avoids the relatively long incision line above the temporal muscle and results only in a barely visible short zigzag scar (Figure 9 (Fig. 9)).

The main advantage of our method lies in the easy planning and in the versatility of the applied vector lines in comparison to the traditional temporal sub-fascial brow lift. The incidence of publications about anchor systems shows a necessity for a reliable technique, if possible, just using re-absorbable simple sutures [29], [30], [31], [32], [33], [34], [35], [36], [37], [38], [39], [40], [41]. In the subcutaneous layers, we expect a far better long time adhesion compared to the sub-fascial plane. The subcutaneous fascial patch is an excellent anchor place to allow for a strong upward pull with simple Vicryl sutures, thus avoiding problems arising from very slow re-absorbable or non-absorbable materials. The third positive aspect is a safe preparation strictly above the temporal branch of the facial nerve [42], [43], [44].

## Notes

### Competing interests

The authors declare that they have no competing interests.
